# Age-Dependent Morphologic Alterations in the Outer Retinal and Choroidal Thicknesses Using Swept Source Optical Coherence Tomography

**DOI:** 10.1371/journal.pone.0159439

**Published:** 2016-07-28

**Authors:** Ichiro Maruko, Hisaya Arakawa, Hideki Koizumi, Reiko Izumi, Hiromi Sunagawa, Tomohiro Iida

**Affiliations:** From the Department of Ophthalmology, Tokyo Women’s Medical University School of Medicine, Shinjuku, Tokyo, Japan; International University of Health and Welfare, JAPAN

## Abstract

**Purpose:**

To evaluate the age-dependent morphologic alterations in the outer retina and choroid at the macula using swept-source optical coherence tomography (OCT).

**Methods:**

Thirty eyes (30 normal subjects; average age, 49 years) were examined; five (age range, third-eighth decades of life) had refractive errors of ±2 diopters or less and no fundus abnormalities. An Early Treatment Diabetic Retinopathy Study (ETDRS) map of the outer retinal and choroidal thickness was constructed using swept-source OCT. The outer retinal and choroidal segmentation lines were drawn automatically, partially manually, within 6 millimeters of the macula.

**Results:**

The mean outer retinal and choroidal thicknesses in the 6-millimeter-diameter circle were 145±13 and 236±68 microns, respectively. The choroidal thickness and age were negatively (r = -0.66, *P*<0.01) correlated; the outer retinal thickness and age were not correlated (r = -0.16, *P* = 0.39). The outer retinal and choroidal thicknesses in the ETDRS map were not correlated (r = -0.13, *P* = 0.49) within 1 millimeter but correlated (r = 0.32, *P*<0.01) within 6 millimeters.

**Conclusions:**

The choroid thins with aging. The outer retina remains stable. Outer retina and choroid are correlated in the entire macula except for the center. ETDRS map can be useful for evaluation of the morphologic relationship between the outer retina and choroid.

## Introduction

While about 80% of the choroidal circulation is ocular blood flow, [1–3] it has not attracted attention since it does not directly cause abnormalities in visual function. However, choroidal observation has become easy using optical coherence tomography (OCT). [4–10] The choroids also are correlated with the ocular axial length and aging. However, the outer retina is one of the most important components of visual function because it includes the photoreceptor cells. Recent OCT studies have reported a relationship between the ellipsoidal zone (inner/outer segment junction [IS/OS]) or interdigitation zone (third line) of the outer retina and visual function. [11,12] Whereas the choroidal circulation nourishes the outer retina, only a few studies have reported on the morphologic relationship between the outer retina and choroid. [13] The current study retrospectively evaluated the relationship between the thicknesses of the outer retina and choroid in each generation of normal subjects.

## Materials and Methods

This study followed the tenets of the Declaration of Helsinki. The institutional review board of Tokyo Women’s Medical University School of Medicine approved this retrospective study that included OCT observation of eyes with macular and retinal disorders (even including normal subjects). We explained the condition of the eye with normal status, and the verbal informed consent was obtained about the usage of images for the academic meetings or reports after anonymization.

Thirty eyes of 30 normal subjects (14 men, 16 women; average age, 49 years) were examined. An Early Treatment Diabetic Retinopathy Study (ETDRS) map (6x6 millimeters) was constructed for the outer retinal and choroidal thicknesses using the volume scan (12x9 millimeters) on swept-source OCT (DRI-OCT, Topcon, Japan). Five cases with ±2 diopters or less refractive errors and no fundus abnormalities were chosen randomly from each generation in their third to eighth decades of life in our normal database. All areas were divided into nine sectors according to the ETDRS grid map style. The whole retina, the outer retina and choroid were defined as the area from inner limiting membrane (ILM) to the inner surface of the retinal pigment epithelium (RPE), the area from the inner border of the outer plexiform layer (OPL) to the inner surface of the RPE and the area from the outer surface of the RPE to the chorioscleral interface, respectively. The segmentation lines of ILM, RPE and choroid except for the OPL were drawn automatically within 6 millimeters of the macula. If some lines were misaligned, we adjusted the lines manually. The segmentation line of the superior border of the OPL was drawn manually in all slices of all cases. Three co-authors (IM, RI, HS) checked these misalignments and drew the superior border of the OPL from about 170 images (about 35μm intervals) obtained from each subject.

When the segmentation lines were checked and modified by the attached software, the thickness in the 6-millimeter ETDRS map was calculated and divided automatically into nine parts (a 1-millimeter-diameter circle at the center, four inner-quarter annuluses between the 1- and 3-millimeter-diameter circles, and four outer-quarter annuluses between the 3- and 6-millimeter-diameter circles). Therefore, the area ratio of the average thicknesses of the outer retina and the choroid in the 6-millimeter-diameter circles was calculated as follows:

1-millimeter circle: inner annulus:outer annulus = 1: 8: 27
1-millimeter circle: 4 x inner-quarter annuluses:4 x outer-quarter annuluses = 1: 8: 27
1-millimeter circle: an inner-quarter annulus: an outer-quarter annulus = 1: 2: 27/4

If the center of the ETDRS map was not aligned with the foveal center, it was manually sifted to the correct location, thinnest point of the whole retina, using attached software. The correlation between each of the outer retinas and choroids in the entire 6-millimeter-diameter circle, the 1-millimeter-diameter circle at the center, the inner annulus between the 1- and 3-millimeter-diameter circles, and the outer annulus between the 3- and 6-millimeter-diameter circles were calculated. The relationship between whole retina and choroid was also calculated. The correlation was considered significant when *P*<0.05. Multiple linear regression analysis was performed to evaluate the correlation between the outer retinal and choroidal thicknesses and age.

## Results

**Fig 1:**
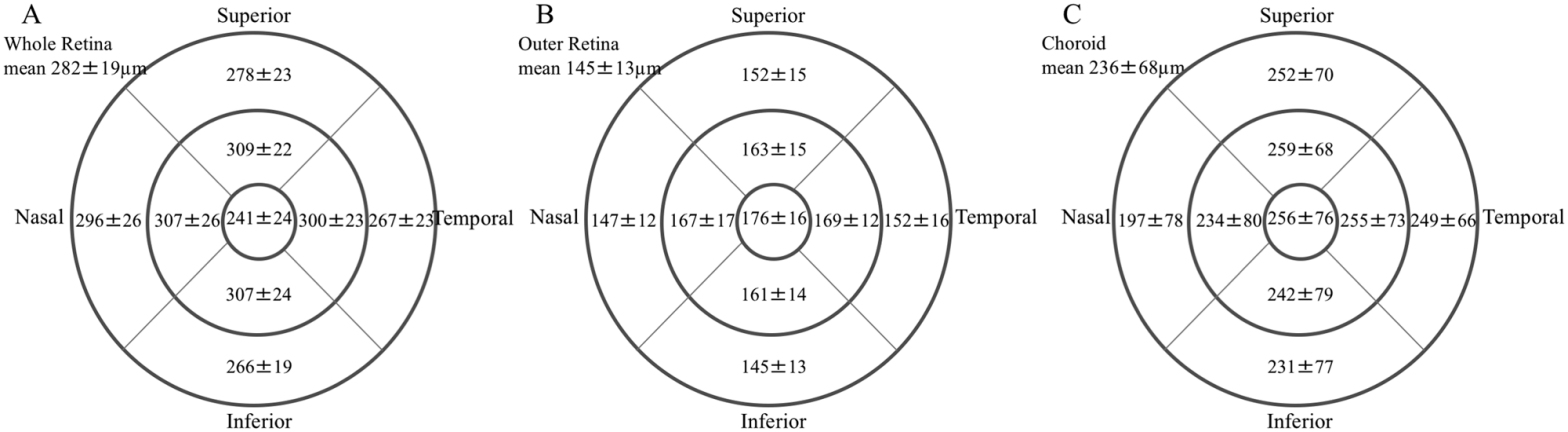
The Early Treatment Diabetic Retinopathy Study (ETDRS) map (6x6 millimeters) of the whole retinal, outer retinal and choroidal thicknesses in normal subjects. The ETDRS map is divided into nine parts (1-millimeter-diameter circle at the center, four inner-quarter annuluses between the 1- and 3-millimeter-diameter circles, and four outer-quarter annuluses between the 3- and 6-millimeter diameter circles). **A, B, and C:** All data are expressed as the mean thickness ± standard deviation of the whole retina, outer retina and choroid, respectively.

**Fig 2:**
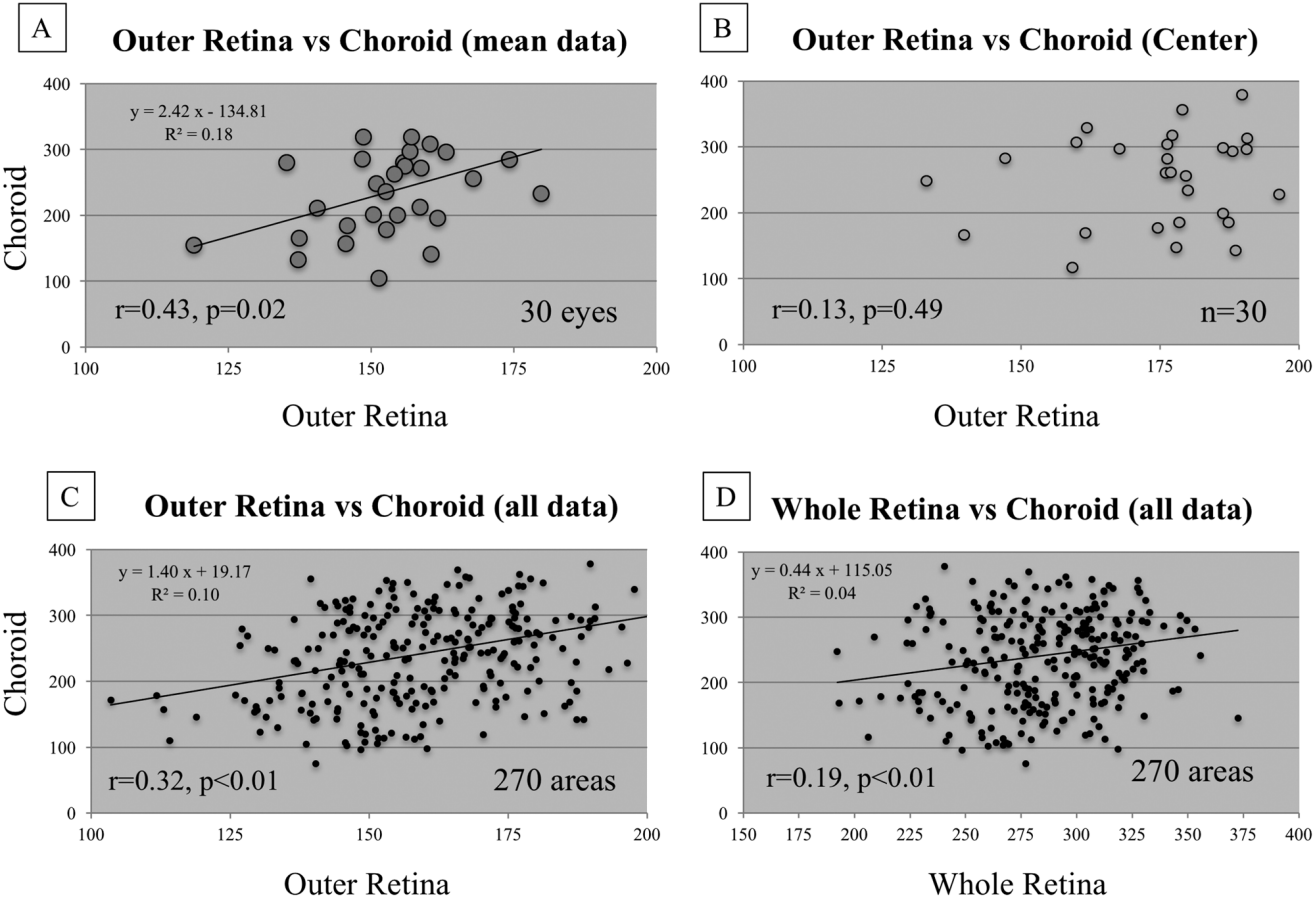
The relationship between the choroidal and outer retinal thicknesses in normal subjects. **A:** There is a significant correlation between the outer retina and choroidal thicknesses in the mean data in the entire 6-millimeter area. **B, C:** The thicknesses of the outer retina and choroid in each area are not correlated within 1 millimeter but are correlated within 6 millimeters. D: The thicknesses of the whole retina and choroid in each area are little correlated within 6 millimeters.

**Fig 3:**
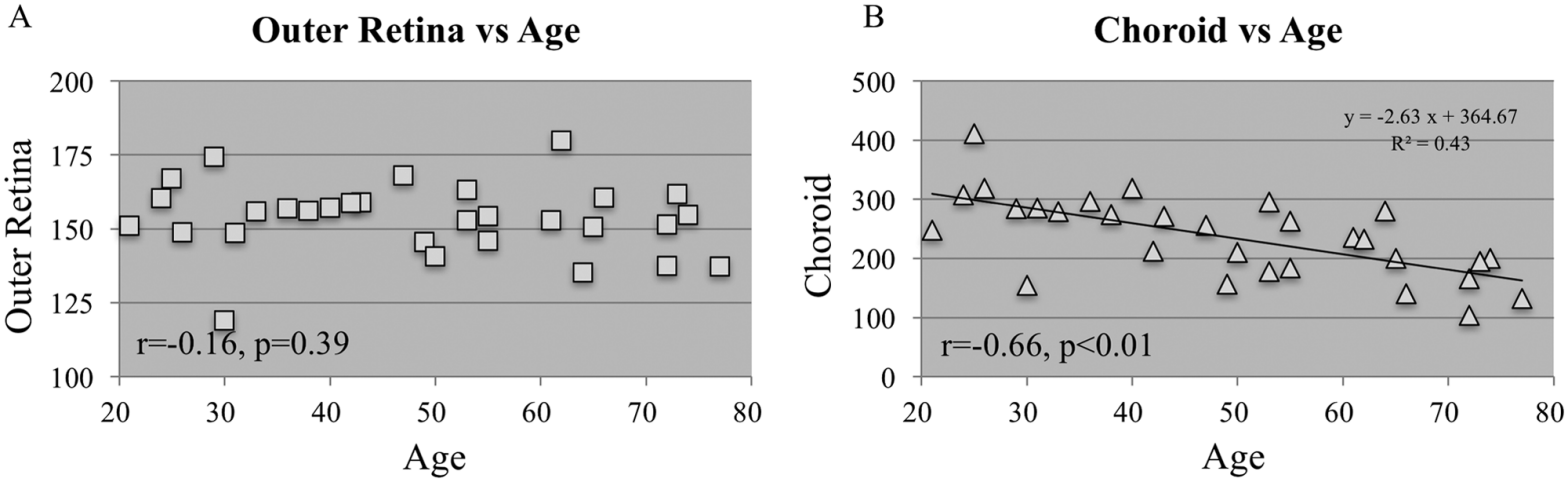
The relationship between thickness and age. **A:** There is no correlation between the thickness of the outer retina and the age. **B:** A negative correlation is seen between the choroidal thickness and age.

The mean whole retina, outer retinal and choroidal thicknesses within the 6-millimeter circle were 282±19, 145±13 and 236±68 microns, respectively (Fig 1). The outer retinal and choroidal thicknesses in subjects in the third to eighth decades of life are shown in Table 1. Considering the mean data from the entire 6-millimeter area, there was a significant correlation between the outer retina and choroidal thicknesses (30 eyes, r = 0.43, *P* = 0.02) (Fig 2A). While the outer retinal thickness was almost unchanged during each decade, the choroidal thickness decreased gradually. In particular, the mean outer retinal thicknesses in the third and eighth decades of life, 160±11 and 148±11 microns, respectively; were similar. However, the choroidal thicknesses were 314±60 and 159±41 microns, respectively; the value in the eighth decade of life was significantly (p<0.01) thinner compared to the value during the third decade. The correlations were observed not by decade of life but by age. While there was no correlation (r = -0.16, *P* = 0.39) between the outer retinal thickness and age (Fig 3A); a negative correlation (r = -0.66, *P*<0.01) was found between the choroidal thickness and age (Fig 3B).

**Table 1:** Whole retinal, Outer Retinal and Choroidal thicknesses in each generation.

Age	Whole Retina (μm)	Outer Retina (μm)	Choroid (μm)
20–29	288± 9	160±11	314±60
30–39	280±28	147±16	257±58
40–49	277±14	158± 8	243±61
50–59	285±21	151± 9	226±51
60–69	284±26	156±16	218±52
70–79	279±17	148±11	159±41

The thicknesses of the outer retina and choroid in each area of the ETDRS map were not correlated (30 areas, r = -0.13, *P* = 0.49) within 1 millimeter (Fig 2B) but were correlated within 3 and 6 millimeters (150 areas, r = 0.30, *P*<0.01; 270 areas, r = 0.32, *P*<0.01, respectively) (Fig 2C). There was little correlation between the whole retina and choroidal thicknesses within 6 millimeters (270 areas, r = 0.19, *P*<0.01) (Fig 2D).

When the correlations were observed by quarter annuluses, the outer retinal and choroidal thicknesses were correlated (r = 0.43, *P* = 0.02) in the upper sector of the inner annulus and the upper (r = 0.49, *P*<0.01), nasal (r = 0.38, *P* = 0.04), and lower (r = 0.44, *P* = 0.01) sectors of the outer annulus. When the inner and outer annuluses were combined and seen as four quarter annuluses between the 1- and 6-millimeter-diameter circles, the outer retinal and choroidal thicknesses were correlated in the upper (r = 0.45, *P*<0.01), lower (r = 0.35, *P*<0.01), and nasal (r = 0.37, *P*<0.01) sectors; in other words, correlations were seen in all sectors except temporally (r = 0.20, *P* = 0.12).

The multiple regression equation used was:

Choroidal thickness = 51.8–2.45 x Age + 1.98 x Outer retinal thickness (Table 2)

**Table 2:** Multiple Linear Regression Analyses for Choroidal Thickness by Age and Outer Retinal Thickness.

Factor	Coefficient	*P* Value	SE
Outer Retinal thickness (μm)	1.98	0.01	0.72
Age (years)	-2.45	<0.01	0.52
Intercept	51.8	0.67	1180

SE = spherical equivalent.

R = 0.745, R2 = 0.56.

Multiple regression equation: subfoveal scleral thickness = 51.8–2.45 × Age + 1.98 × Outer Retinal thickness

Multiple regression analysis showed that the choroidal thickness was well determined by the outer retinal thickness and age (R = 0.745, R^2^ = 0.56).

Outer retinal thickness = 118.3 + 0.20 x Age + 0.11 x Choroidal thickness (Table 3)

**Table 3:** Multiple Linear Regression Analyses for Choroidal Thickness by Age and Choroidal Thickness.

Factor	Coefficient	*P* Value	SE
Choroidal thickness (μm)	0.11	0.01	0.04
Age (years)	0.2	0.23	0.16
Intercept	118.3	<0.01	15.8

SE = spherical equivalent.

R = 0.477, R2 = 0.23.

Multiple regression equation: subfoveal scleral thickness = 118.3 + 0.20 × Age + 0.11 × Choroidal thickness

Multiple regression analysis showed that the choroidal thickness was well determined by the outer retinal thickness and age; however the coefficient of determination was moderate (R = 0.477, R^2^ = 0.23).

## Discussion

The relationships between the outer retinal and choroidal thicknesses in normal eyes were evaluated by the thickness map at the macular area. As a result, while the choroid thinned with increasing age, the thickness of the outer retina remained stable. In addition, whereas the outer retinal and choroidal thicknesses were correlated somewhat in the entire macula, no correlation was seen between them within the 1-millimeter-diameter circle, including the central fovea.

The outer retina includes the OPL, outer nuclear layer (ONL), external limiting membrane (ELM), and retinal photoreceptor layer, and it is certainly related to visual function. Diseases such as retinitis pigmentosa or other retinal dystrophies are characterized by thinning of the ONL at the location of the photoreceptor nuclear bodies, and the decreased number of photoreceptor cells themselves can be associated partly with the thinning of the ONL. [14–16] The ELM is the series of aligned junctions between the photoreceptors and the outermost end of the Müller cells, or support cells, of the retina, and the ELM is thought to be disrupted or disappear as a result of retinal damage. The ellipsoidal zone of the photoreceptor inner segment, formerly referred to as the IS/OS line, is thought to be related closely to visual function. The ellipsoidal zone often becomes unclear or is not visible on OCT in patients with visual loss because it includes photoreceptors such as cone opsin and rhodopsin. When fluid pools in the subretina in rhegmatogenous retinal detachments, [17] age-related macular degeneration, [18–20] or central serous chorioretinopathy, [21,22] even if the anatomy of the retina returns to normal after treatment, the visual acuity does not improve unless the line recovers. The interdigitation zone, previously referred to as the third line, is thought to be the villus of the RPE and/or the photoreceptor outer segment tips. The anatomic part in which this line is located has not been determined; however, it is also thought to be related to visual function. [23–26] While each layer of the outer retina has its own function, oxygen and nutrients are supplied to all layers by the choroidal circulation, so these should be affected by choroidal abnormalities. However, only a few reports have evaluated the morphology of the outer retina and choroid.

Although morphologic evaluation of the choroids previously was possible only with relatively invasive examinations such as indocyanine green angiography until recently, observation of the choroids, especially measurement of the thickness using OCT, has become a recent topic of interest. Much research on the choroids using OCT has shown that they become thicker in eyes with central serous chorioretinopathy or Vogt-Koyanagi-Harada disease and become thin in eyes with high myopia. The choroidal thickness also decreases with age even without any disease processes. Because the choroidal circulation is thought to account for about 80% of the ocular circulation, [1–3] the effect of the choroid on the outer retina is immeasurable. Although Karahan et al. [13] reported a correlation between the outer retinal and choroidal thicknesses at several points within the macular area in normal eyes, only the thickness of the temporal area reached significance. Moreover, it is not fully understood because in many cases in which some patients have high myopia or are an advanced age the visual acuity is maintained even with extremely thin choroids. In such situations, we three-dimensionally evaluate the relationship between the outer retinal and choroidal thicknesses not only in the central fovea but also in the macular area using the ETDRS map.

In the current examination of normal eyes, there was a correlation between the outer retinal and choroidal thicknesses in the whole area of 6 millimeters of the macular area. By contrast, there was little correlation between the whole retina and choroid; in other words, the outer retina became thinner as the choroid became thinner. This result seems reasonable because the choroid nourishes the outer retina. On the other hand, the outer retina did not have the correlation with increasing age in the multiple regression equation analysis although the choroid had the correlation with the age and the outer retina. Therefore unlike the expectation that the outer retina and the choroid would become thinner with increasing age, the thickness of the outer retina was maintained. Also, the choroidal thickness rarely affected the thickness of the outer retina around the central fovea. These results may indicate the presence of a mechanism in the outer retina at the central fovea that maintains the condition of the outer retina to protect the visual function. In the current study, it was difficult to see how far this mechanism could maintain the homeostasis of the outer retina at the central fovea since no cases with extremely thin or pathological layers were included. Future studies that include patients with various diseases should be undertaken.

The current study identified a correlation between the outer retina and choroid in the entire macular area by reevaluating three-dimensional scans of each generation of normal eyes on SS-OCT and examining their morphologic relationship. In contrast, interestingly, no such correlation was seen in the central fovea and the outer retina was preserved.

The current study had some weaknesses including its retrospective nature, a small number of subjects, only normal eyes, and the fact that the morphologic choroidal thickness does not completely reflect the choroidal circulation itself. However, the current study emphasized the importance of considering the morphologic relationship between the outer retina and choroid. SS-OCT is considered effective because it can observe all retinal layers to the choroid around the entire macular area simultaneously.
